# A Case of HELLP Syndrome in a Patient with Immune Thrombocytopenic Purpura

**DOI:** 10.1155/2010/692163

**Published:** 2010-09-13

**Authors:** Sebastián Ben, Fabián Rodríguez, Carlos Severo, Natalia Debat

**Affiliations:** Department “A” of Obstetrics and Gynecology, University of the Republic School of Medicine, Pereira-Rossell Hospital, Felipe Contucci 3890, 11700 Montevideo, Uruguay

## Abstract

We will describe the clinical case of a pregnant patient with chronic Immune Thrombocytopenic Purpura who develops preeclampsia syndrome with HELLP syndrome. These concomitant and independent conditions become complex, resulting in thrombocytopenia which creates diagnostic, prognostic and therapeutic inconveniences.

## 1. Introduction

Immune Thrombocytopenic Purpura (ITP) is a disease that is present in approximately 1-2 of every 10,000 pregnancies characterized by the formation of autoantibodies which after binding to platelet antigens, destroy the platelets in the reticuloendothelial system, mainly in the spleen, causing thrombocytopenia [1]. There would be a decrease in the production of platelets in the bone marrow due to autoantibodies [2]. These autoantibodies are normally lgGs that recognize platelet membrane glycoproteins, with Glycoprotein (GP) llb/lIla and GP lb-IX being the most frequent ones [3]. When the evolution of the disease lasts beyond a period of six months, it is called “chronic” and can be primary or secondary to other pathologies such as HIV or malignant diseases.

Antiplatelet autoantibodies are found in 60%–70% of the cases [4]. It is a nonspecific characteristic since it appears in other pathologies such as preeclampsia-eclampsia syndrome, HELLP syndrome, gestational thrombocytopenia, antiphospholipid syndrome, and even in normal pregnancies [5].

ITP diagnosis is of exclusion. There are no pathognomonic components of this pathology. According to the American College of Obstetrics and Gynecology (ACOG), there are some elements traditionally associated with this disease such as: (1) persistent thrombocytopenia (platelet count < 100 × 10^9^/L with or without megakaryocytes in peripheral smear), (2) normal or increased medullary megakaryocytes, (3) exclusion of other systematic diseases or drugs that are associated with thrombocytopenia, and (4) absence of splenomegaly [5] (Table 1). 

The preeclampsia-eclampsia syndrome is determined by the presence of hypertension in pregnancy with a 24-hour count of albuminuria > 0.3 g. An important form of severe preeclampsia is the HELLP syndrome. This syndrome was first described by Weinstein in 1982. HELLP is an English acronym that stands for Hemolysis, Elevated Liver Enzymes and Low Platelets. There are diagnostic criteria such as the Tennessee Classification: evidence of hemolysis with LDH > 600 Ul/L or greater or bilirubin 1.2 mg/dL or greater, hepatic dysfunction with AST > 70 Ul/L or greater, and platelets < 100 × 10^9^/L or less. It is considered complete or true if it contains these three components and incomplete if it has only two. There is a classification of severity such as the Mississippi Classification that divides HELLP syndrome into three classes according to the degree of thrombocytopenia, considering it mild when between 150 × 10^9^/L and 100 × 10^9^/L, moderate when between 100 × 10^9^/L and 50 × 10^9^/L, and severe when <50 × 10^9^/L [6].

## 2. Case Report

The patient is a 19-year-old female with chronic ITP since the age of nine and who is under treatment with oral Prednisone, 30 mg/day. This treatment is not followed regularly. She is G2, A1 and is admitted to the Pereira-Rossell Hospital with a 29-week pregnancy due to painful uterine contractions that subside spontaneously with rest while hospitalized. For 48 hours and every 12 hours, an i.m. dose of 6 mg Dexamethasone was administered with the purpose of reducing the occurrence of neonatal respiratory distress syndrome.

The patient had severe thrombocytopenia episodes during the 14, 22, and 26 weeks of gestation, being treated with Immunoglobulin G, Gamma globulin and Methylprednisolone, respectively.

**Figure 1:**
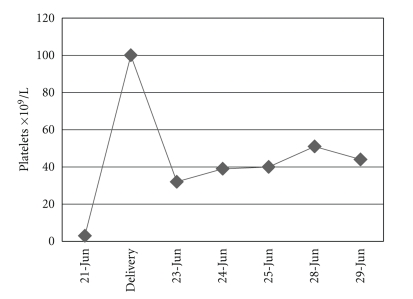
Platelet count.

**Figure 2:**
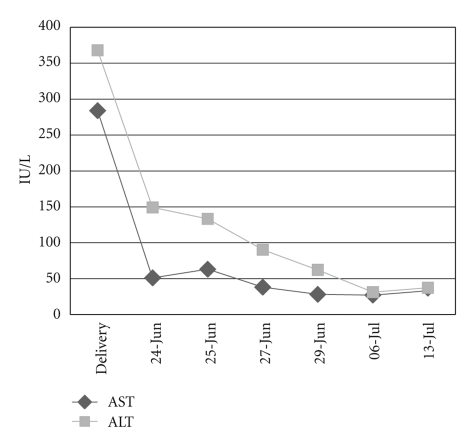
Aminotransferases.

While hospitalized, she was hypertensive with levels of 150/100 mm Hg., accompanied with ecchymoses on right buttock with no petechiae, and had a platelet count of 3 × 10^9^/L. The transaminase values showed AST: 284 IU/L and ALT: 362 IU/L; LDH: 1,280 U/L. The 24-hour albuminuria test is of 0.48 g. Serum creatinine and hematocrit levels were normal. One g/kg of weight of Immunoglobulin G was administered. At the same time, she spontaneously started labor. Eight concentrates of platelets were administered, showing a rise up to 100 × 10^9^/L (Figure 1). The vaginal delivery was carried out without hemorrhagic complications, giving birth to a live newborn weighing 1,142 g, with an Apgar score of 4/6, showing good evolution and presenting no thrombocytopenia. For 24 hours the patient received an i.v. dose of 1/g/hour magnesium sulphate. She remaind normotensive under treatment with a dose of 500 mg alpha methyldope every eight hours. She was given 50 mg/day of oral Prednisone. The value of liver enzymes decreased returning to normal during the first days of puerperium (Figure 2).

**Figure 3:**
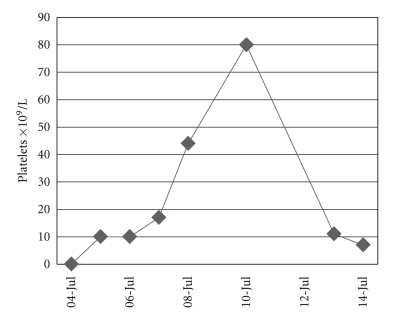
Platelet count.

On July 4 (day 12 of puerperium), the patient presented epistaxis and ecchymoses on one thigh, and her platelet count was < 5 × 10^9^/L (Figure 3). Six platelet concentrates were administered, and a three-day treatment with an i.v. dose of 1 g/day Methylprednisolone was started. Later, an oral dose of 40 mg/day Dexamethasone was given for four days and, finally, 40 g/day of oral Prednisone. An i.v dose of 2,500 mcg Anti-D Immunoglobulin and two i.v. doses of 2 mg Vincristine were administered weekly. Initially, the patient responded well to the treatment. On day 21 of puerperium, the patient started a spontaneous epistaxis and ecchymoses with a platelet count of 5 × 10^9^/L. Six platelet concentrates were given. On day 23 of puerperium, a splenectomy was done by laparotomy. The postoperative evolution was good, and the patient was discharged from the hospital on day seven of the treatment with oral Prednisone.

## 3. Discussion

The concomitance of chronic ITP, preeclampsia-eclampsia syndrome, and HELLP syndrome is extremely rare. In January 2010, we did a bibliographical search in PubMed and Cochrane Database of Systematic Reviews using the following key words: HELLP Syndrome, Idiopathic Thrombocytopenic Purpura, Pregnancy, Autoimmune Diseases and PreEclampsia. No similar cases were reported. 

The platelet count normally decreases during pregnancy although it is uncommon to have a count below 100 × 10^9^/L. In our case, there were three episodes of severe thrombocytopenia that required hospitalization and treatment. In this case, there was no neonatal thrombocytopenia even knowing that the antiplatelet antibodies belonged to the lgG variety which are able to pass through the placenta and cause fetal and neonatal thrombocytopenia [5]. Neonatal thrombocytopenia is present in approximately 25% of the cases, being severe (< 50 × 10^9^/L) in about 10% of the cases. Approximately 4% of neonates show thrombocytopenia (< 20 × 10^9^/L). Acute hemorrhagic complications such as intracranial hemorrhage is a rare event observed in less than 1% of the cases [1, 7–9]. Since fetal platelet destruction is produced in the reticuloendothelial system, the prematurity such as the case we are describing could be a protective factor for fetal compromise, because due to the immaturity of the reticuloendothelial system, such compromise could be less serious. It is not possible to predict the risk of fetal or neonatal thrombocytopenia based on the maternal platelet count, the presence of antiplatelet antibodies, or previous splenectomy [1, 5, 10].

The preeclampsia was diagnosed because the patient was hypertensive with 24-hour albuminuria values of 0.48 g. The liver function test alterations and the thrombocytopenia correspond to the diagnosis of HELLP syndrome.

As to the mode of delivery, there is no evidence that the performance of a C-section diminishes the risk of neonatal intracranial hemorrhage, increasing, in a significant manner, maternal hemorrhagic risks; therefore, it is being recommended that the mode of delivery be determined by the obstretic conditions [1, 5]. The British Society of Hematology recommends that thrombocytopenia counts of 50 × 10^9^/L are safe for a vaginal delivery, and 80 × 10^9^/L are safe for a C-section and spinal or epidural anesthesia [1]. Nonsteroidal anti-inflammatory drugs should not be prescribed as analgesics.

During pregnancy, ITP treatment depends on the risk of maternal hemorrhage and on having safe values of platelets at the moment of delivery [5]. The increase in the platelet count happens in 70% of the cases with the administration of Prednisone [5]. In the case we are describing, a treatment with Prednisone was carried out with an oral dose of 30 mg/day that the patient did not follow adequately.

The i.v. immunoglobulin G, 1 g/kg of weight, was administered before delivery with a platelet count of 3 × 10^9^/L. According to recommendations by the ACOG, this procedure would be appropriate for cases of ITP with a platelet count lower than 10 × 10^9^/L in the third trimester, lower than 30 × 10^9^/L associated with bleeding, or as preoperative or prior to delivery [5]. Its effect is observed between six and seventy-two hours after administering the medication. This effect remains for two to three weeks with a positive response in 80% of the cases [1].

The transfusion of platelets is reserved for cases of vital risk when there are acute hemorrhages or when it is time for delivery, as was our case, or surgery [5].

Another therapeutical option used was the administration of i.v. Methylprednisolone, 1 g/day, for three days. After that, the patient was given an oral dose of 40 mg/day Dexamethasone for four days, which has shown to be effective as initial treatment in ITP in adults, with a remission rate of 40%.

During puerperium, a dose of 50 mcg/kg of weight Anti-(Rh) D Immunoglobulin was administered. It is a suitable treatment for nonsplenectomized Rh (D)-positive patients [1]. A weekly i.v. dose of 2 mg Vincristine was administered twice. This drug has shown a temporary increase in the platelet count in two thirds of the patients with ITP, but there is a lasting remission in about 10% of the patients. Vincristine has adverse effects such as neutropenia, fever, and phlebitis [1].

The splenectomy was done on day 23 of the puerperium because of the unsatisfactory response to the treatment. The performance of a splenectomy during pregnancy may be technically difficult due to the size of the uterus after a 20-week pregnancy and may cause spontaneous abortion during the first trimester. If it were necessary to perform this surgery during pregnancy, the ideal moment would be in the second trimester. A splenectomy is associated with a complete remission of the thrombocytopenia in ITP in about 66% of the patients [5]. Despite this, it does not predict better neonatal results in future pregnancies. There may be a maternal clinical improvement, but there is not necessarily an immunological remission.

## Figures and Tables

**Table 1:** Causes of thrombocytopenia during pregnancy [5].

Gestational thrombocytopenia
Hypertension induced by pregnancy
HELLP syndrome
Immune Thrombocytopenic Purpura
HIV infection
Systemic lupus erythematosus
Antiphospholipid syndrome
Hypersplenism
Disseminated intravascular coagulation
Thrombotic thrombocytopenic purpura
Hemolytic uraemic syndrome
Congenital thrombocytopenias
Medications (heparin, zidovudine, quinine, sulphonamides)
Pseudothrombocytopenia
